# Personality and occupational correlates of anxiety and depression in nurses: the contribution of role conflict, core self-evaluations, negative affect and bullying

**DOI:** 10.1186/s40359-022-00921-6

**Published:** 2022-09-10

**Authors:** Zahra Hosseini, Atefeh Homayuni

**Affiliations:** 1 Social Determinants in Health Promotion Research Center, Hormozgan Health Institute, Hormozgan University of Medical Sciences, Bandar Abbas, Iran; 2 Hormozgan University of Medical Sciences, Bandar Abbas, Iran

**Keywords:** Bullying, Self-evaluation, Affect, Conflict, Anxiety, Depression, Nurses

## Abstract

**Background:**

The work environment and the fatiguing nature of nursing are risk factors that cause psychological disorders. This study was conducted with the aim of investigating the relationship between role conflict, core self-evaluations, negative affect, and bullying with anxiety and depression in nurses.

**Methods:**

This cross-sectional study was conducted on the nurses working in hospitals of Bandar Abbas, Iran over 4 months (August 2019–November 2019). Data were collected using the Positive and Negative Affect Schedule scale, Role Conflict Questionnaire, Core Self-Evaluations Scale, Negative Acts Questionnaire-Revised, Beck Depression Inventory-Second Edition (BDI-II) and Beck Anxiety Inventory. Data were analyzed using t-test, one-way analysis of variance, Pearson correlation coefficient and multiple regression analysis using SPSS version 22 software and the significance level was considered 0.05.

**Results:**

The findings revealed that there was a significant positive relationship between role conflict (r = 0.422, *p* < 0.01), negative affect (r = 0.715, p < 0.01), and bullying (r = 0.443, *p* < 0.01) with anxiety. Moreover, there was a significant negative relationship between core self-evaluations with anxiety (r = − 0.482, *p* < 0.01). Also, the findings indicated that there was a significant positive relationship between role conflict (r = 0.382, *p* < 0.01), negative affect (r = 0.672, *p* < 0.01), and bullying (r = 0.433, *p* < 0.01) with depression. There was a significant negative relationship between core self-evaluations and depression (r = − 0.603, *p* < 0.01). Moreover, regression analysis results revealed that negative affect, role conflict, and core self-evaluations predicted 54.3% of anxiety variance significantly. And lastly, negative affect and core self-evaluations predicted 53.3% of depression variance significantly.

**Conclusions:**

Findings indicated that having high negative affect, low core self-evaluations, high role conflict, and exposure to bullying at work enhances the rate of depression and anxiety in nurses. Hence, it is essential to improve the mental health of nurses and thus the quality of care provided by them through recognizing suitable supportive strategies and interventions.

## Introduction

Mental health, as one of the most key constituents of a healthy life, tries to lessen negative emotions such as anxiety, depression, and hopelessness and avoid the incidence of morbid symptoms in individuals [1]. Anxiety and depression are two of the most prevalent health problems in today's society [2]. Occupation is one of the factors that influence mental health. Employment, irrespective of finance, and satisfying some basic human requirements such as self-esteem, can be a source of stress and influence the physical and mental health. Nurses are among the people who are always at risk of injuries caused by stress and anxiety. World Health Organization has regarded this occupation among the hard occupations, because of the continuous exposure of this occupational group to stress and anxiety [3].

The results of the research conducted by Angermeyer et al. [4] showed that nurses are among the employed groups that are exposed to stress and psychological issues. Depression, anxiety, and fatigue are prevalent complications in this group. Wang et al. [5] in their study found that workplace risk factors, such as pain, sorrow and death of the patient, conflict with physician and coworkers, hard work, sensitive working conditions, chemicals, devices, and various disinfectant and carcinogenic materials related to the workplace are the causes of stress, depression, and anxiety. The findings of different studies indicate that the prevalence of depressive symptoms among nurses in the United States was between 35 and 41%, 11–80% in Iranian nurses, 35% in Chinese nurses, 17% in Australian midwives, and 51% in Brazilian nurses. Besides, approximately 33% of French nursing managers and 10% Canadian nurses were suffering from depressive symptoms. Also, investigations have revealed a high prevalence of anxiety in nursing professionals. The findings demonstrate that the prevalence of anxiety ranges from 20% in Australian midwives to 32–43% in Chinese nurses, 40–46% in Iranian nurses, and 44–66% in Brazilian nurses [6].

The role of numerous personal and organizational factors in the development of these mental disorders has been confirmed. Role conflict is one of the organizational variables and role stressors that has been shown to decrease the productivity of employees and increase their levels of anxiety and depression. Role conflict refers to the employee's perception of more than one set of specific demands that result in a discrepancy between the manager’s and the employee's understanding of suitable job performance conditions [7]. Tarrant and Sabo [8] in their study among nurse executives indicated that there is a moderate positive relationship between role conflict and depression. The results of a cross-sectional study conducted by Yoshizawa et al. [9] on psychiatric nurses in Japan showed that subjects with higher scores for role conflict were more likely to display depressive symptoms. Berthelsen et al. [10] in their study on Norwegian nurses found that role conflict as a work factor was significantly associated with follow-up symptoms of anxiety and depression.

Among the personality traits that can enhance vulnerability to anxiety and depression are core self-evaluations and negative affect. Core Self-Evaluations (CSE) is an essential evaluation that represent individuals' fundamental assumptions and evaluations about their own worth and competence. Core self-evaluations can be viewed as a broad latent structure that can be recognized by at least four characteristics: self-esteem, locus of control, generalized self-efficacy, and neuroticism [11]. Studies have revealed that among self-evaluations and self-cognitions of individuals, self-esteem (one of the components of CSE) is a significant factor that influences the onset and development of depression, and there is a significant negative correlation between CSE and depression in adolescents and managers [12]. The researchers found that CSE was perhaps a key predictor of less physical and mental health issues and more rehabilitation. Besides, researchers found that CSE was a key predictor of positive coping strategies during unemployment, better health, and fewer psychological issues (such as anxiety, stress, and depression) among unemployed adults [13].

Watson and Tellegen [14] found that positive and negative affect have different associations with indicators of mental health, including anxiety and depression. Investigations have revealed that positive excitements and emotions may improve an individual’s resistance to negative occasions and inhibit the incidence of mental and even physical disorders. Mood traits "negative affect" and "emotion regulation" are the leading elements of theoretical models explaining the development of anxiety and depressive disorders. Negative affect is a temperamental factor most commonly associated with anxiety and other emotional disorders. Negative affect has been considered as a fundamental common characteristic of these disorders as well-as a well-established risk factor for their development [15].

Experiencing bullying in the workplace is another factor that can result in anxiety and depression in nurses who have been the target of bullying in the workplace. It is expected that individuals who are exposed to bullying at work are suffering more from physical-psychological (i.e., anxiety, depression, burnout, and post-traumatic stress disorder), psychological (i.e., stress, sleep disorders, aggression, distraction, low self-esteem, and absence of self-confidence) and psychosocial (i.e., sleep issues, headaches, stomach disorders, difficulty in concentrating, emotional exhaustion, absence of courage, high levels of anger and memory issues) problems [16]. Recognizing that a person cannot cope with a persistent stress stimulus can result in negative health problems. Workplace bullying can be regarded as a serious stressful stimulus, in particular, if an individual had concluded that he/she cannot manage the persistent stressful situation of bullying, and incompatible responses appear in the form of psychological and physical symptoms [17].

According to what mentioned above, it can be said that personality characteristics and different occupational factors may interact in the onset and development of depression and anxiety. In Iran, most studies have studied the prevalence of anxiety and depressive disorders in nurses [18–21]. In few studies, the contribution of both personality and occupational factors in prevalence of anxiety and depression have been examined [22–24]. Furthermore, the physical and mental health of nurses are associated with the quality of their performance in providing care for patients, their job satisfaction and their job productivity. The first step in creating effective interventions to promote nurses' mental health is to identify the relevant and predictive personality and occupational factors. Therefore, the study was developed to respond to this question: 1. Are there relationships between personality traits (negative affect and core self-evaluations) and occupational factors (role conflict and bullying) with anxiety and depression?

The following hypotheses are proposed:

1.There are significant differences in anxiety/depression mean scores according to the demographic variables (gender, marital status, years of experience, age groups, education level and ward of affiliation) [23, 25–27]; 2. There is a significant positive relationship between role conflict and anxiety/depression among nurses [8–10]; 3. There is a significant positive relationship between negative affect and anxiety/depression among nurses [28, 29]; 4. There is a significant positive relationship between bullying and anxiety/depression among nurses [17, 30]; 5. There is a significant negative relationship between core self-evaluations and anxiety/depression among nurses [12, 13]; 5. Role conflict, negative affect, core self-evaluations and bullying predict anxiety/depression among nurses [17, 31].

## Materials and methods

### Sampling procedure

This study was based on a cross-sectional design. The statistical population of this study included nurses working in private and public hospitals in Bandar Abbas, south of Iran.

According to the previous study [32], the bullying rate was 31%. Sample size was calculated using the following formula: n = the minimum required sample size, z = level of confidence (1.96), p = parameter for sample calculation (31%), d = margin of error (0.05).

$$n = \frac{{z_{{1 - {\raise0.5ex\hbox{$\scriptstyle \alpha $} \kern-0.1em/\kern-0.15em \lower0.25ex\hbox{$\scriptstyle 2$}}}}^{2} pq}}{{d^{2} }} = \frac{{\left( {1.96} \right)^{2} \times \left( {0.31} \right) \times \left( {0.69} \right)}}{{\left( {0.05} \right)^{2} }} = 329$$

Based on this formula, a sample size of 329 anticipated for the study.

Due to the limited statistical population and the need for more detailed information, all nurses were studied through census method. The participants were selected from both public and private hospitals (including 8 hospitals) and from different wards of affiliation (children, adults, intensive care unit (ICU), internal ward, thalassemia and dialysis, coronary care unit (CCU), neonatal intensive care unit (NICU), neurology, operating room and maternity ward) and different work shifts (morning, evening and night). Inclusion criteria were: 1) being employed as a nurse in hospital for at least one year and 2) willingness to participate in the study. On the other hand, the exclusion criteria were: 1) not having enough time to participate in the study, 2) less than 1 year's nursing experience.

After obtaining the required permission from the university and institutional consent from hospitals' managements, one of the researchers referred to the hospitals to collect data. The researcher explained about the aim of the study and how the participants can fill out the questionnaires. They were informed that their information will remain confidential with the researcher and written consent was obtained from the participants. The questionnaires were given to nurses who volunteered to participate in the study.

### Measures

We used self-administered questionnaires to collect the data. The following tools were used to measure variables and collect data:

1. *Negative Acts Questionnaire-Revised* The 22-item Negative Acts Questionnaire-Revised (NAQ-R) [33] was used to determine the frequency of exposure to bullying behaviors within the last 6 working months. This questionnaire measures 3 categories of harassing acts including: work-related bullying (7 items, such as: Being ordered to do work below your level of competence), person-related bullying (12 items, such as: Being exposed to an unmanageable workload) and physical intimidation (3 items, such as: threats of violence or physical abuse or actual abuse). On this questionnaire, participants are asked to indicated their responses on a 5-point Likert scale from 1(never) to 5 (daily). The lowest score is 22 and the highest score is 120. This scale has good internal consistency, as its internal consistency coefficients ranged from 0.88 to 0.90 [33]. In the present study, Cronbach's alpha coefficient for this questionnaire was 0.92.
2. *Role Conflict Questionnaire* Role conflict was measured by 8 questions, taken from Rizzo et al.'s factor analysis [34]. The scale measured the degree of incongruity of expectations associated with a role (e.g. "I have to do things that should be done differently under different conditions). The answers are determined on a 4-point Likert scale from "very correct" (1) to "not correct at all" (4). Overall scores ranged from 8 to 32 points whereby the higher score indicated the lower role conflict. In the present study, Cronbach's alpha coefficient for this questionnaire was 0.87.
3. *Anxiety Questionnaire* To measure anxiety, Beck Anxiety Inventory (BAI) [35] was used. This scale contains 21 terms, each of which describes a common symptom of anxiety. The subject is requested to determine the extent to which each of the symptoms, on a 4-point scale varying from 0 to 3, has caused distress and annoyance in the past month. Questionnaire’s internal consistency coefficient is 0.92, its reliability by a one-week interval retest method is 0.75 and the correlation of its questions ranges from 0.3 to 0.76 [36, 37]. In the present study, Cronbach's alpha coefficient for this questionnaire was 0.94.
4. *Negative Affectivity* To assess the negative affect, the negative affectivity (NA) scale from the well-known PANAS instrument was employed [38]. The scale has 10 items. Participants are asked to indicate the extent to which they experienced particular negative emotions on a 5-point Likert scale ranging from "very low, not at all" (score 1) to "very high" (score 5). Overall scores for each subscale was 10–50 points. Internal compatibility coefficient is 0.87 for the negative affect subscale [38]. In the present study, Cronbach's alpha coefficient for this questionnaire was 0.91.
5. *Depression Questionnaire* To assess depression, Beck Depression Inventory-Second Edition (BDI-II) was used. BDI-II is a self-reported index of depressive symptoms experienced in the past 2 weeks [39]. The test items include a total of 21 items associated with the different symptoms that the subjects must answer on a 4-point scale from 0 to 3. The minimum score in this test is 0 and the maximum is 63. Because of the importance of this tool in diagnosing clinical intervention, numerous psychometric investigations have been done on its psychometric features. Beck et.al [39] found that its validity coefficient by the retest method according to the distance between the times of application and the type of test population ranged from 0.48 to 0.86. In the present study, Cronbach's alpha coefficient for this questionnaire was 0.91.
6. *Core Self-Evaluations Scale (CSES)* Core self-evaluations was measured with a 12-item inventory [40]. This scale involves statements about typical thoughts/feelings and behaviors (e.g. "I am confident to get the success I deserve in life"). These items are answered on a 5-point Likert scale, ranging from 1 (strongly disagree) to 5 (strongly agree). Items 2, 4, 6, 8, 10 and 12 were reversely scored. A higher score means a higher core self-evaluations. Judge et al. [40] obtained the reliability of this questionnaire 0.78. In the present study, Cronbach's alpha coefficient for this questionnaire was 0.72.

### Ethical consideration

The present research was approved by the ethics committee of Hormozgan University of medical sciences (#IR.HUMS.REC.1397.093). The participants were informed that participation in the study was voluntary and they had the right to withdraw at any time during the data collection process. Written informed consent was obtained from all participants.

### Data analysis

Data were analyzed in SPSS version 22 statistical software. Means and standard deviation values ​​were calculated for continuous variables. Differences between the groups were tested by t-test and one-way analysis of variance (ANOVA). Pearson correlation analysis was conducted to explore the relationship between variables. We used the criteria introduced by Cohen et al. [41] to assess the strength of correlation coefficients (0 < 0.30 = weak; 0.30 <|r|< 0.50 = moderate; 0.50 <|r|< 0.70 = strong; 0.70 <|r|< 1 = very strong). Results of the Kolmogorov–Smirnov test showed that there was a normal distribution of the anxiety and depression data (*p* value > 0.05). To assess multicollinearity issues, the variance inflation factor (VIF) was calculated and the value of all variables were in the acceptable range. Multiple regression was used to assess the ability of predictor variables in predicting depression and anxiety. We entered the predictors in the regression equation based on the theoretical importance of the variables according to the evidence in the literature [16, 29, 42–45]. The level of significance was considered to be 95% (*p* < 0.05).

## Results

The participating nurses’ demographic information showed that 12.5% of the participants were male and 87.5% were female. 73.8% nurses were married and the majority of nurses held a Bachelor’s degree (84.5%). 43.9% nurses were between the ages of 30–39 years. 38.6% participants had more than 10 years’ experience of nursing service.

### Group differences

The results of t-test showed that there was a significant difference between the mean BAI scores (df = 319, t = 2.5, *p* < 0.05) and BDI-II scores (df = 319, t = 2.47, *p* < 0.05) in male and female nurses. Mean scores on BAI and BDI-II were significantly higher for male than female nurses (Table 1).

**Table 1:** Compare the mean scores of anxiety and depression by gender

Variable	Male	Female	T	df	*p*-value
BAI scores	14.62 ± 11.51	10.81 ± 10.02	2.5	319	0.013
BDI-II scores	10.8 ± 9.2	8.05 ± 7.38	2.47	319	0.014

One-Way Anova results showed that there were not significant differences in BAI and BDI-II mean scores according to marital status and years of experience, but nurses in different age groups and wards experienced varying amounts of anxiety and depression. Nurses who were 40 years of old or higher, experienced more anxiety and depression. Nurses who worked at ICU and children wards experienced more anxiety and depression, respectively. In addition, nurses who have diploma experienced more depression (Table 2).

**Table 2:** One-Way Anova results to compare the mean scores of anxiety and depression in terms of demographic variables

Variable		BAI scores	BDI-II scores	BAI scores		BDI-II scores	
		Mean (SD)		F	*p*-value	F	*p*-value
Marital status	Single	10.34 ± 9.01	9.77 ± 7	1.14	0.32	0.49	0.61
	Married	11.23 ± 11.14	8.06 ± 7.75				
	Divorced	9.4 ± 8.26	8 ± 6.7				
Education level	Diploma	15.42 ± 12.62	13 ± 8.26	2.41	0.07	3.65	0.01
	Associate degree	15.66 ± 6.02	6.33 ± 2.08				
	Bachelor’s degree	11.02 ± 10.56	8.42 ± 7.74				
	Master’s degree and higher	8.76 ± 7.11	5.33 ± 5.07				
Years of experience	< 3 years	11.3 ± 9.41	9.82 ± 7.18	1.39	0.24	0.65	0.58
	3–5 years	10.53 ± 9	8.73 ± 7.48				
	5–10 years	11.06 ± 10.07	8 ± 7.26				
	> 10 years	12.1 ± 10.92	8.61 ± 7.6				
Age group	20–29 years	11.07 ± 10.23	9.02 ± 7.63	4.31	0.01	3.23	0.04
	30–39 years	10.07 ± 9.29	7.62 ± 6.92				
	≥ 40 years	14.03 ± 12.015	10.03 ± 7.92				
Ward of affiliation	Children	13.61 ± 11.24	9.92 ± 9.73	3.61	0.001	2.53	0.01
	Adults	11.24 ± 8.55	7.45 ± 6.89				
	ICU	14 ± 12	9.51 ± 8.89				
	Internal ward	8.98 ± 6.53	8.73 ± 5.79				
	Thalassemia and dialysis	13.35 ± 9.82	7.29 ± 5.39				
	CCU	7.2 ± 4.94	6.38 ± 4.11				
	NICU	9.85 ± 8.05	9.63 ± 7.5				
	Neurology	12.31 ± 11.5	9.53 ± 7.71				
	Operating room and maternity ward	13.04 ± 11.48	9.6 ± 7.9				

### Associations between personality/occupational variables and anxiety and depression

The mean and standard deviation of the variables, as well as their correlation with each other are presented in Table 3. Results of Pearson correlation indicated that there were moderate positive relationships between RCQ scores (r = 0.42), NAQ-R scores (r = 0.44), and its dimensions with BAI scores (*p* < 0.01). NAS scores had a very strong positive relationship with BAI scores (r = 0.71, *p* < 0.01). Also, there were moderate positive relationships between RCQ scores (r = 0.38), NAQ-R scores (r = 0.43), and its dimensions with BDI-II scores (*p* < 0.01). NAS scores had a strong positive relationship with BDI-II scores (r = 0.67, *p* < 0.01). Besides, there were moderate negative relationship between CSES scores with BAI scores (r = − 0.48) and strong negative relationship between CSES scores with BDI-II scores (r = − 0.6) (*p* < 0.01).

**Table 3:** Descriptive statistics and correlation matrix between study variables

	Variable	Mean	Standard Deviation	1	2	3	4	5	6	7	8	9
1	NAQ-R scores	38.9	13.04	1	0.94^**^	0.86^**^	0.64^**^	0.56^**^	− 0.39^**^	0.47^**^	0.44^**^	0.43^**^
2	PRB scores	18.9	7.39	0.94^**^	1	0.66^**^	0.61^**^	0.52^**^	− 0.38^**^	0.46^**^	0.37^**^	0.41^**^
3	WRB scores	15.9	5.64	0.86^**^	0.66^**^	1	0.36^**^	0.53	− 0.33^**^	0.44^**^	0.44^**^	0.4^**^
4	PI scores	4.24	1.62	0.64^**^	0.61^**^	0.36^**^	1	0.33^**^	− 0.22^**^	0.32^**^	0.25^**^	0.25^**^
5	NAS scores	18.58	7.62	0.56^**^	0.52^**^	0.53^**^	0.33^**^	1	− 0.51^**^	0.42^**^	0.71^**^	0.67^**^
6	CSES scores	42.12	5.42	− 0.39^**^	− 0.38^**^	− 0.33^**^	− 0.22^**^	− 0.51^**^	1	− 0.32^**^	− 0.48^**^	− 0.6^**^
7	RCQ scores	18.55	5.89	0.47^**^	0.46^**^	0.44^**^	0.32^**^	0.42^**^	− 0.32^**^	1	0.42^**^	0.38^**^
8	BAI scores	10.72	10.96	0.44^**^	0.37^**^	0.44^**^	0.25^**^	0.71^**^	− 0.48^**^	0.42^**^	1	0.64^**^
9	BDI-II scores	7.68	8.11	0.43^**^	0.41^**^	0.4^**^	0.25^**^	0.67^**^	− 0.6^**^	0.38^**^	0.64^**^	1

*NAQ-R* Negative acts questionnaire-revised, *PRB* Person-related bullying, *WRB* Work-related bullying, *PI* Physical intimidation, *NAS* Negative affect schedule, *CSES* Core self-evaluations scale, *RCQ* Role conflict questionnaire, *BAI* Beck anxiety inventory, *BDI-II* Beck depression inventory-second edition

^**^p < 0.01

A hierarchical multiple regression ascertained how much variance in BAI scores and BDI-II scores could be accounted for by independent variables (Tables 4 and 5).

**Table 4:** Results of multiple hierarchical regression analysis in predicting BAI scores through predictor variables

	Variables	B	S.E	β	t	*p*-value	R	R^2^	∆R^2^
Model 1	NAS scores	1.02	0.06	0.71	17.77	0 < 001	0.71	0.51	–
Model 2	NAS scores	0.91	0.07	0.64	13.82	0 < 001	0.73	0.53	0.017
	CSES scores	− 0.3	0.09	− 0.15	− 3.29	0.001			
Model 3	NAS scores	0.88	0.07	0.62	11.84	0 < 001	0.73	0.53	0.001
	CSES scores	− 0.29	0.09	− 0.15	− 3.14	0.002			
	NAQ-R scores	0.03	0.04	0.04	0.84	0.401			
Model 4	NAS scores	0.84	0.07	0.59	11.42	0 < 001	0.74	0.54	0.014
	CSES scores	− 0.27	0.09	− 0.13	− 2.92	0.004			
	NAQ-R scores	− 0.01	0.04	− 0.01	− 0.22	0.825			
	RCQ scores	0.26	0.09	0.14	3.003	0.003			

*B* Unstandardized coefficients, *S.E* Standard error, *β* Beta (Standardized coefficients), *R* Coefficient of correlation, *R*^*2*^ R Square, *∆R*^*2*^ R square change

**Table 5:** Results of multiple hierarchical regression analysis in predicting BDI-II scores through predictor variables

	Variables	B	S.E	β	t	*p*-value	R	R^2^	∆R^2^
Model 1	NAS scores	0.72	0.04	0.67	15.92	0 < 001	0.67	0.45	–
Model 2	NAS scores	0.53	0.05	0.49	11.01	0 < 001	0.74	0.55	0.092
	CSES scores	− 0.53	0.07	− 0.35	− 7.85	0 < 001			
Model 3	NAS scores	0.52	0.05	0.48	9.5	0 < 001	0.74	0.55	0.001
	CSES scores	− 0.53	0.07	− 0.35	− 7.67	0 < 001			
	NAQ-R scores	0.02	0.03	0.03	0.58	0.562			
Model 4	NAS scores	0.5	0.05	0.47	9.19	0 < 001	0.74	0.55	0.005
	CSES scores	− 0.51	0.07	− 0.34	− 7.51	0 < 001			
	NAQ-R scores	− 0.00	0.03	− 0.00	− 0.09	0.922			
	RCQ scores	0.12	0.06	0.08	1.86	0.063			

NAS scores was fed in the first step, CSES scores was entered in the second step, NAQ-R scores in the third step and RCQ scores in the fourth step. 51.1% of variances of BAI scores accounted for by NAS scores in step 1 and by CSES scores in step 2 as 1.7%. An additional 0.01% of the variance in BAI scores was explained by the addition of NAQ-R scores in step 3. Finally, RCQ scores accounted for an additional 1.4% variance in BAI scores. The results showed that NAS scores, CSES scores and RCQ scores predicted 54.3% of the total variance in BAI scores.

NAS scores, CSES scores, NAQ-R scores and RCQ scores were entered the regression equation in the first, second, third and fourth step, respectively. 45.5% of variances of BDI-II scores accounted for by NAS scores in step 1 and by CSES scores in step 2 as 9.2%. An additional 0.01% of the variance in BDI-II scores was explained by the addition of NAQ-R scores in step 3. Finally, RCQ scores accounted for an additional 0.5% variance in BDI-II scores. The results showed that NAS scores and CSES scores predicted 55.3% of the total variance in BDI-II scores.

## Discussion

This study aimed to examine the relationship between role conflict, core self-evaluations, negative affect, and bullying with anxiety and depression. Results indicated that there were significant positive relationships between role conflict, negative affect, bullying and its dimensions with anxiety and depression. Besides, there were significant negative relationships between core self-evaluations with anxiety and depression. One of the strengths of the current research was the selection of sample members from numerous hospitals, as well as the assessment of personality factors along with occupational factors as predictors of depression and anxiety.

### The relationship between role conflict with anxiety and depression

This study found that there was a significant positive relationship between role conflict with anxiety and depression. These findings were in consistent with previous studies conducted by Zheng et al. [42], Desouky and Allam [46], Niedhammer et al. [31] and Nakada et al. [47]. Zheng et al. [42] found that female health care and social service providers experienced a high level of depression. Organizational role conflict was significantly and positively associated with female social workers' depression. To explain the relationship between role conflict with anxiety and depression, the stress exchange model can be applied. Instead of a direct association between stressors and mental issues (i.e., the consequences of stress), this model claims that there is a cognitive state between them: evaluation and coping. Workplace stressors are evaluated by individuals, both in primary and secondary evaluations. During the first evaluation, individuals categorize a stimulus as a negative stimulus, or a positive stimulus, or a stimulus not linked to their well-being. Recognizing a stimulus as a negative stimulus is often accompanied by unpleasant excitements or general discomfort. The secondary evaluation includes a more detailed analysis and development of coping approaches. If sufficient resources are not present or unsuitable coping approaches are selected, constant exposure to stressors can result in short-term reactions such as physiological modifications (enhanced heart rate, blood pressure, and hormone levels), reduced performance, failure, anger, and irritability. If stress continues over long periods, short-term reactions convert to long-term reactions to stress, such as mental health issues, anxiety, physical complications, depression, and absenteeism [48].

### The relationship between core self-evaluations with anxiety and depression

In this study it was also demonstrated that core self-evaluations predicted anxiety and depression. The current finding was similar with findings from other studies conducted by Zuo et al.[12], Peláez-Fernández et al.[13], Dou et al. [43] and Hentrich, et al. [44]. Individuals with positive core self-evaluations tend to select and maintain challenging jobs, and are happier with their jobs and lives, report lower levels of stress and conflict, are better capable to adapt to modifications, and take advantage of advantages and opportunities. These people do better in situations that need positive interpersonal relationships with stressful conditions or in circumstances that need enduring stress and have better physical health [11]. They can adapt to external obstacles and experience favorable and beneficial excitements and attitudes, in contrast, people with weak and negative core self-evaluations believe that their actions are useless or that they can do little to improve unfavorable situations, so they are susceptible to experiencing negative excitements and emotions. These individuals consider themselves inferior to others and focus on their failures and deficiencies and consider themselves victims of the environment [49]. People with lower levels of CSE look at life negatively and passively, look at events as threatening instead of opportunity and perceive the world as unmanageable. These tendencies, when a person is faced with specific events, result in enhanced stress and perception of stressors in them, and consequently result at the beginning of the depression and anxiety in these individuals [12].

### The relationship between negative affect with anxiety and depression

The results showed that there was a significant positive relationship between negative affect with anxiety and depression. This finding was in consistent with previous studies conducted by Thomas et al. [29], Tortella-Feliu et al. [15], and Cohen et al. [28]. Negative affect is a common underlying factor in both anxiety disorders and depression [50]. Indeed, depression has long been characterized by high negative and low positive affect, and persistent miserable moods and reduced pleasing experiences are the leading properties of this disorder. On the other hand, anxiety has principally been defined by high negative affect and high physiological stimulation [28]. Individuals with high levels of negative emotions experience irritating moods and negative excitements and tend to put themselves in circumstances that are faced with more stressors. Negative affect is related to mental complaints, anxiety, and low ability to cope with pressure and stress, and individuals with high negative emotions are anxious, worried, and low in energy, respond to situations with anxiety, fear, worry, and bitterness [51, 52]. The incidence and existence of negative emotions such as hopelessness, despair, sorrow, and grief cause a person to feel out of control over the condition, apply more ineffectual coping approaches, feel less competent to their ability to face life, and that makes him feel a kind of constant weakness. Consequently, the incidence of negative affect decreases psychological well-being and enhances the occurrence of psychological distress.

### The relationship between bullying with anxiety and depression

The results indicated that there was a significant positive relationship between bullying with anxiety and depression. This finding was similar with findings from studies conducted by Presti et al. [53], Duru et al. [16], Yun and Kang [17], Ruiz et al. [45] and Minton et al. [30]. Presti et al. [53] found that exposure to workplace bullying was positively associated with an increased presence of anxiety and depression. Bullying is a very severe form of psychosocial risk that is often identified as a predictor of depression. The relationship between bullying and depression can be explained using the "Stress-as-Offense-to-Self" theory. Based on this theory, bullying is particularly stressful as it directly threatens the self. Bowling and Beehr (2006) claim that bullying is a threat to a person's self-image and can result in depression. This relationship between bullying and depression has been proven in some occupations and workplaces, in nurses, municipal workers, hospital staff, and specific non-industrial sample [54]. In addition, "cognitive activation theory of stress", recommends that constant exposure to occupational stressors through continuous activation mechanisms damages and disrupts health. For instance, it is likely that the exposure to bullying, because of the prolonged physiological and cognitive activation of the hypothesis related to anxiety and trying to cope with that condition, activates a rise in feelings of anxiety and fatigue [55].

### Multiple relationships between independent variables with anxiety and depression

Finally, the hierarchical regression analysis showed that negative effect, core self-evaluations and role conflict can significantly predict anxiety. Also, negative affect and core self-evaluations can predict depression. The possible explanation for these findings is that the individuals with low and negative core self-evaluations and high negative affect, report higher levels of stress and conflict. These individuals tend to put themselves in circumstances that are faced with more stressors and in these situations, they have a low ability to cope with pressure and stress. When they faced with job stressors such as role conflict, they feel out of control over the condition and use ineffective coping strategies. As a result, they experience more negative and unpleasant emotions such as anxiety and depression.

## Research limitations

The current investigation has a cross-sectional nature. Thus, researchers can not draw causal inferences according to their findings. The nurses in the research were selected by a convenience sampling method, so the generalizability for other samples is not fully guaranteed. Findings of the current study, as in many other investigations, may encourage participants to use approaches based on gaining social approval and avoiding notoriety because of the use of self-report tools instead of the study of actual behavior. In other words, because of the sensitivity of the bullying subject, many people may have rejected to answer the bullying questionnaire honestly. Consequently, it is recommended that researchers in future studies use behavioral observation.

## Conclusions

Relying on the results of this study, hospital managers can take various measures to promote the mental health of nurses. These measures can be focused on the work environment, occupational factors or personality traits of nurses. Workplace-focused interventions included decreasing job stressors such as role conflict, employing anti-bullying policies in the hospital, and the development of a reporting system for all occasions associated with bullying in the hospital. Managers also can seek help from the psychologists to hold training courses to enhance nurses' core self-evaluations (increase their self-esteem and self-efficacy and decrease their neuroticism) and decrease their negative affect.

## Data Availability

The datasets generated and analyzed during the current study are not publicly available due to confidentiality and privacy related issues but are available from the corresponding author on reasonable request.

## Abbreviations

NAQ-R: Negative acts questionnaire-revised
CSES: Core self-evaluations scale
PANAS: Positive and negative affect scale
BAI: Beck anxiety inventory
BDI-II: Beck depression inventory-second edition
